# The Association of Regular Dog Walking With Mobility, Falls, and Fear of Falling in Later Life

**DOI:** 10.1093/gerona/glaf010

**Published:** 2025-01-11

**Authors:** Eleanor Gallagher, Amanda Lavan, Rose Anne Kenny, Robert Briggs

**Affiliations:** Mercer’s Institute of Successful Ageing, St James’s Hospital, Dublin, Ireland; Discipline of Medical Gerontology, Trinity College Dublin, Dublin, Ireland; Mercer’s Institute of Successful Ageing, St James’s Hospital, Dublin, Ireland; Discipline of Medical Gerontology, Trinity College Dublin, Dublin, Ireland; Discipline of Medical Gerontology, Trinity College Dublin, Dublin, Ireland; The Irish Longitudinal Study on Ageing, Trinity College Dublin, Dublin, Ireland; Discipline of Medical Gerontology, Trinity College Dublin, Dublin, Ireland; The Irish Longitudinal Study on Ageing, Trinity College Dublin, Dublin, Ireland; (Medical Sciences Section)

**Keywords:** Gait, Older, Pet, Physical Activity

## Abstract

**Background:**

It has been suggested that dog walking may protect against falls and mobility problems in later life, but little work to date has examined this. The aim of this study was to assess if regular dog walking was associated with reduced likelihood of falls, fear of falling, and mobility problems in a large cohort of community-dwelling older people.

**Methods:**

Participants ≥60 years at Wave 5 of The Irish Longitudinal Study on Ageing were included. Regular dog walking was ≥4 days/week by self-report. The control group consisted of participants who did not own a dog or who did not regularly walk their dog. Falls and fear of falling were self-reported. Mobility was measured with Timed-Up-and-Go (TUG). Logistic regression models assessed associations between regular dog walking and outcomes of interest.

**Results:**

Regular dog walkers (629/4 161, 15%) had a significantly faster TUG (10.3 (10.1–10.5) versus 11.7 (11.1–12.2) seconds, *t* = 2.11, *p* = .0343) and a lower likelihood of unexplained falls (OR 0.60 (0.38–0.96; *p* = .034), fear of falling (OR 0.79 (95% CI 0.64–.98); *p* = .032), and mobility problems (0.64 (0.45–0.91); *p* = .015) in fully adjusted models. Regular dog walking was also associated with a significantly lower likelihood of fear of falling (OR 0.79 (95% CI 0.64–0.98); *p* = .032).

**Discussion:**

This study demonstrates a significantly lower prevalence of mobility impairment, falls, and fear of falling among community-dwelling older people who regularly walk their dogs. Although longitudinal and dedicated studies are required, older people should be encouraged to continue regular dog walking where feasible, as it may help in maintaining mobility and reducing falls.

## Graphical Abstract

**Graphical Abstract:**
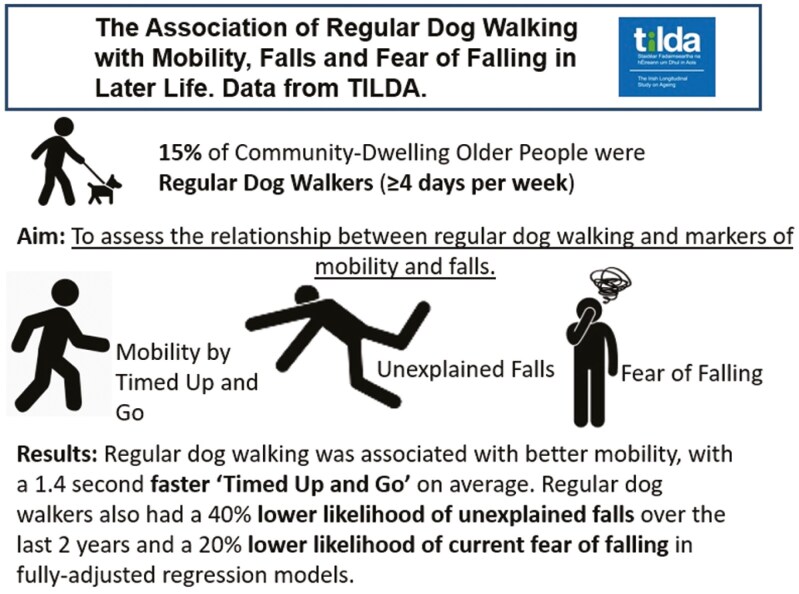

## Background

Falls can have a profound effect on well-being, quality of life, and functional independence in later life. They represent the most common cause of accidental death of older people (1) and the most frequent reason for presentation to hospital (2). Falls, particularly fall causing injury, significantly increase the likelihood of nursing home admission, cognitive decline, fear of falling, and early mortality (3–5).

Appropriate engagement with physical activity confers important health benefits for older people, including reduction of functional decline, improved quality of life, and better mood (6,7). Engaging in physical activity in late life has also been shown to reduce the risk of falls (8) and is protective against mobility loss and the development of fear of falling (9).

Dog ownership may act as an important driver for physical activity in later life by providing support and motivation to walk (10), with dog encouragement of walking and a sense of dog-walking obligation identified as important factors encouraging physical activity (11). It has been suggested that regular dog walking may protect against falls and mobility problems in later life, but little work has been done to date examining this.

The aim of this study therefore was to assess whether community-dwelling older people who regularly walk their dog had reduced likelihood of falls, fear of falling, and mobility problems.

## Method

### Study Design

This cross-sectional study examined the association between regular dog walking and falls, fear of falling, and mobility in a large cohort of community-dwelling people aged ≥60 years, utilizing data from the Irish Longitudinal Study on Ageing (TILDA).

The TILDA study design has been outlined previously (12). Briefly, there are 3 components to data collection: a computer-assisted personal interview carried out by social interviewers in the participants’ own home; a self-completion questionnaire completed and returned by the participant; and a comprehensive center-based health assessment or a modified home-based health assessment carried out by trained research nurses. Study waves are conducted at 2-yearly intervals. We used data from TILDA Wave 5 in this study, collected from 2018 to 2019. Wave 5 was the first wave where data collection on dog ownership and dog walking were included in the study.

### Dog Walking

Participants were asked if they had a pet and those that responded positively were then asked how frequently they walk their dog. Regular dog walking was defined as ≥4 days/week.

The control group was therefore either participants who did not have a dog or who had a dog but did not engage in regular dog walking. A post hoc analysis was conducted to examine outcomes specifically in the group of participants who had a dog but did not walk them regularly.

### Falls

Incidence of falls in the last 2 years was elicited by self-report. Explained falls were those caused by a simple slip/trip as per the participants, whereas unexplained falls were not caused by slips or trips and had no apparent cause.

Fear of falling was defined as answering “yes” to: “Are you afraid of falling?”

### Mobility

Mobility was measured with the Timed-Up-and-Go (TUG) (13). The TUG was measured in the participants’ own homes using an available chair, which varied in height and design (assessors were instructed to find a chair that matched the chair used in the TILDA health assessment as closely as possible). During the TUG, the participant stands up from a chair, walks 10 feet/3 meters at a normal pace, turns, and walks back to the chair at a normal pace before sitting again. The time taken from the command “Go” to when the participant was sitting again with his/her back resting against the back of the chair was recorded using a stopwatch. Mobility impairment was defined as a TUG of > 15 seconds (14).

### Covariates

Covariates were chosen a priori based on the likelihood of modifying the relationship between dog walking and falls/mobility. Covariates were age, sex, marital status (married, separated/divorced, widowed, never married), highest level of educational attainment (primary, secondary, tertiary), body mass index, alcohol excess (by CAGE score with score >2 indicating alcohol excess), heart disease (self-report of angina, myocardial infarction, heart failure or arrhythmias), prior stroke, polypharmacy (prescribed ≥5 medications), the number of days of moderate physical activity per week by self-report and chronic disease burden (the number of the following medical comorbidities reported by the participant: chronic lung disease, eye problems [cataracts, age-related macular degeneration, glaucoma], cancer, osteoporosis, chronic liver disease, arthritis, diabetes, and Parkinson’s disease).

### Statistical Analysis

Chi-square tests were used for comparison of binary variables and proportions with 95% confidence intervals were estimated. Mean comparison/*t*-tests were used to assess differences in continuous variables across groups, reporting the *t*-statistic, as well as the mean/95% confidence interval and *p*-value. Logistic regression models reporting odds ratios assessed the association between regular dog walking and binary outcomes of interest (mobility impairment, falls, and fear of falling), adjusting for age, sex, marital status, educational attainment, alcohol excess, heart disease, stroke, polypharmacy, and chronic disease burden. Logistic regression models were also used to assess the association between dog ownership without regular dog walking and outcomes of interest, with regular dog walkers excluded from these analyses. A *p*-value < .05 was considered statistically significant.

### Ethics

The TILDA study was approved by the Faculty of Health Sciences Research Ethics Committee at Trinity College Dublin and all participants gave informed written consent. All experimental procedures adhered to the Declaration of Helsinki.

## Results

Of the 4 161 participants (mean age 71 years; 54% female), 15% were regular dog walkers.

Table 1 demonstrates the characteristics of the study sample (at TILDA Wave 5) by regular dog walking. Regular dog walkers were younger with lower rates of polypharmacy and heart disease, and more likely to have never smoked. They were also more likely to engage in moderate physical activity each week.

**Table 1.** Study Sample Characteristics by Dog Walking Status

	Regular Dog Walkers *n* = 629	Not Dog Walker *n* = 3,532
Mean age, years (95% CI)	68.1 (67.6–68.7)	71.2 (70.9–71.4) ^**^
Female (prop. with 95% CI)	0.55 (0.51–0.59)	0.54 (0.52–0.56)
Educational attainment: (Prop with 95% CI)		
-Primary	0.22 (0.19–0.26)	0.23 (0.21–0.24)
-Secondary	0.40 (0.36–0.43)	0.39 (0.37–0.41)
-Tertiary	0.38 (0.35–0.42)	0.38 (0.37–0.40)
Marital status: (prop with 95% CI)		
-Married	0.72 (0.68–0.75)	0.68 (0.66–0.69)
-Never married	0.08 (0.06–0.11)	0.08 (0.07–0.09)
-Separated/divorced	0.06 (0.05–0.09)	0.07 (0.07–0.08)
-Widowed	0.13 (0.11–0.16)	0.17 (0.16–0.18)
Alcohol excess (prop with 95% CI) ^A^	0.10 (0.08–0.13)	0.08 (0.07–0.09)
Body mass index (prop with 95% CI)		
- < 25.0	0.37 (0.33–0.41)	0.38 (0.37–0.41)
-25.0–30.0	0.42 (0.38–0.46)	0.41 (0.39–0.43)
- > 30.0	0.21 (0.18–0.25)	0.21 (0.19–0.22)
Physical activity (prop with 95% CI) ^B^		
-0 days	0.51 (0.47–0.54)	0.54 (0.53–0.56)
-1–3 days	0.19 (0.16–0.22)	0.21 (0.20–0.22)
-4–7 days	0.31 (0.27–0.35)	0.25 (0.23–0.26) ^*^
Smoking history: (prop with 95% CI)		
-Never	0.41 (0.38–0.45)	0.47 (0.46–0.49)
-Past	0.46 (0.43–0.50)	0.43 (0.41–0.45)
-Current	0.12 (0.10–0.15)	0.09 (0.08–0.10)
Heart disease (prop with 95% CI) ^C^	0.09 (0.07–0.11)	0.12 (0.11–0.13) ^*^
Chronic disease number: (prop with 95% CI) ^D^		
-0	0.43 (0.39–0.47)	0.36 (0.34–0.38) ^*^
-1	0.35 (0.31–0.39)	0.37 (0.36–0.39)
-2	0.21 (0.18–0.24)	0.26 (0.24–0.27)
-≥3	0.01 (0.01–0.02)	0.01 (0.01–0.01)
Polypharmacy (prop with 95% CI) ^E^	0.27 (0.23–0.30)	0.35 (0.33–0.36) ^**^

*Notes:* CI = confidence interval; prop = proportion.

Regular dog walking defined as walking dog 4 or more times per week.

^A^Alcohol excess defined as score > 2 on Cut Down, Annoyed, Guilty, Eye Opener (CAGE) scale;

^B^self-reported number of days with moderate physical activity per week;

^C^heart disease defined as self-report of heart attack, angina, heart failure, or arrhythmia;

^D^chronic diseases included are diabetes, Parkinson’s disease, chronic lung disease, arthritis, liver disease, osteoporosis, cancer and eye disease (glaucoma, cataracts, age-related macular degeneration [ARMD]);

^E^polypharmacy defined as prescribed ≥5 regular medications.

^*^Denotes *p* < .05 for comparison with chi-square test;

^**^denotes *p* < .01 for comparison by chi-square test.

### Mobility

**Figure 1.**
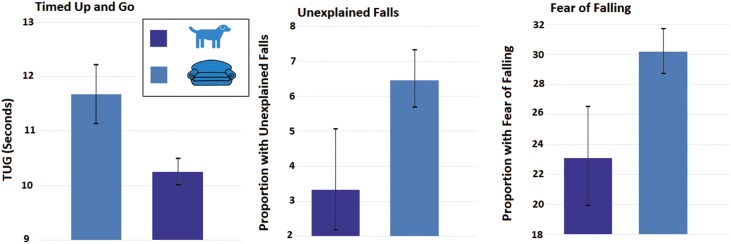
Mobility and fall by dog walking status. Data presented are mean TUG in seconds with 95% confidence interval and the proportion of participants with unexplained falls and fear of falling with a 95% confidence interval. Regular dog walking was defined as walking a dog ≥4 days/week, by self-report. Unexplained falls are falls not caused by slips or trips over the last 2 years, by self-report. Fear of falling is defined as answering “yes” to “Are you afraid of falling?” TUG = Timed Up and Go.

Regular dog walkers had a significantly faster TUG than nondog walkers (10.3 (95% CI 10.1–10.5) vs 11.7 (95% CI 11.1–12.2) seconds) (*t* = 2.11; *p* = .0343). See Figure 1.

The proportion of participants with mobility impairment (TUG > 15 seconds) was 0.05 (95% CI 0.03–0.07) in the regular dog walking group and 0.11 (95% CI 0.10–0.12) in nondog walkers.

**Figure 2.**
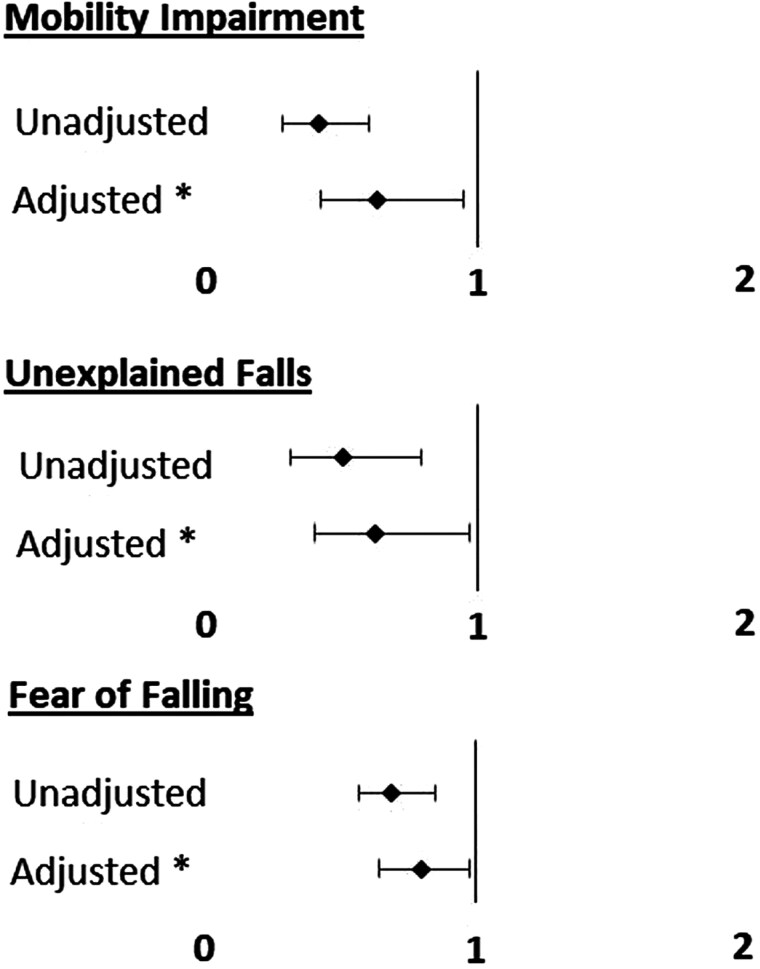
Logistic regression models presenting odds ratios with regular dog walking as a predictor of mobility and falls outcomes. Data presented are output from logistic regression models (reporting odds ratios with 95% confidence intervals) with regular dog walking as a predictor of mobility/falls outcomes. Model adjusted for age, sex, marital status, educational attainment, alcohol excess, heart disease, stroke, polypharmacy, and chronic disease burden. Regular dog walking was defined as walking a dog ≥4 days/week mobility impairment defined as Timed up and Go >15 seconds. Unexplained falls are falls not caused by slips or trips over the last 2 years, by self-report. Fear of falling is defined as answering “yes” to “Are you afraid of falling?”

As shown in Figure 2, logistic regression models demonstrated that regular dog walking was associated with a lower likelihood of mobility impairment in fully-adjusted models (odds ratio 0.64 (95% CI 0.45–0.91); *p* = .015).

### Falls

The proportions of participants with explained falls were similar across groups (0.21 (95% CI 0.18–0.24) in the regular dog walkers, compared with 0.19 (95% CI 0.18–0.20) in the nondog walkers; *X*^2^ = 0.846, *p* = .358)).

As shown in Figure 1, there was a significantly higher proportion of participants with unexplained falls in the nondog walking group (0.06 (95% CI 0.06–0.07) vs 0.03 (95% CI 0.02–0.05); *X*^2^ = 9.218; *p* = .002). In fully adjusted logistic regression models, regular dog walkers had a significantly lower likelihood of unexplained falls (odds ratio 0.60 (95% CI 0.38–0.96; *p* = .034). See Figure 2.

The proportion of participants with fear of falling was significantly higher in the nondog walking group (0.30 (95% CI 0.29–0.32) vs 0.23 (0.20–0.27); *X*^2^ = 13.150, *p* < .001)). See Figure 1. In fully adjusted models, regular dog walking was associated with a significantly lower likelihood of fear of falling (OR 0.79 (95% CI 0.64–0.98); *p* = .032). See Figure 2.

### Post Hoc Analysis: Dog Owners Who Did Not Regularly Walk Their Dog

Almost 13% of the study sample (525/4 161) owned a dog but did not engage in regular dog walking. Compared with participants who regularly walked their dog, this group was younger (68.9 (95% CI 68.3–69.6) vs 68.1 (95% CI 67.6–68.7); *p* = .0466), with higher rates of polypharmacy (30% vs 23%, *X*^2^ = 8.0075, *p* = .005), more likely to be married (79% vs 72%, *X*^2^ =14.2580, *p* = .003) and were more likely to be a never-smoker (49% vs 41%, *X*^2^ = 6.2700, *p* = .044). There were no differences in sex distribution, educational attainment, rates of alcohol misuse, heart disease, and chronic disease burden when compared with the group who engaged in regular dog walking.

The mean number of days spent engaging in moderate physical activity per week was also similar (2.29 (95% CI 2.08–2.51) in regular dog walkers compared with 2.14 (1.91–2.37) in dog owners who did not regularly walk their dog; *p* = .3307).

Having a pet dog but not engaging in regular dog walking was not associated with a lower likelihood of mobility impairment (odds ratio 0.95 (95% CI 0.68–1.33) in fully adjusted models.

Having a pet dog but not engaging in regular dog walking was not associated with unexplained falls (odds ratio 0.88 (95% CI 0.58–1.34) but was associated with a higher likelihood of fear of falling (odds ratio 1.40 (95% CI 1.28–1.73) in fully adjusted models.

## Discussion

This study, utilizing data from Wave 5 of the population-representative TILDA study, demonstrates that 15% of community-dwelling older people are regular dog walkers (≥4 times per week) and that regular dog walking is associated with better mobility, with 1.4 seconds faster TUG on average (15). Regular dog walkers also had a 40% lower likelihood of unexplained falls over the last 2 years and a 20% lower likelihood of current fear of falling in fully-adjusted regression models.

Previous studies have demonstrated the health benefits of increased physical activity by regular dog walking. As well as increased exercise (10,11), regular dog walking may also be associated with lower body mass index, fewer sitting events during the day, an improved cardiovascular risk factor profile, and a lower risk of cardiovascular disease (15–17).

Regular dog walking may also have further benefits beyond simply increasing physical activity, which could impact mobility and fall risk, for example, by increasing a sense of community, as a source of social support, and as a means of increasing social interaction (18,19). During the coronavirus disease 2019 (COVID-19) pandemic, regular dog was also shown to be an important factor in protecting against loneliness among older people (20). It is important to note that we found broadly similar levels of reported physical activity between regular dog walkers and nondog walkers in this study.

To the best of author’s knowledge, this is the first study to examine the association of dog walking with mobility performance and falls in a large cohort of community-dwelling older people. A prior study of almost 400 dog walkers did demonstrate a faster walking speed among dog walkers, however (21), although a further study of 637 older people found that the decline in gait speed was slower among pet owners (22). Little work to date has examined the relationship among dog walking, falls, and fear of falling in later life.

Post hoc analysis demonstrated that although having a pet dog but not engaging in regular dog walking was not associated with falls or mobility impairment, it was associated with a higher likelihood of fear of falling. Fear of falling, and the avoidant behavior associated with it, may therefore underpin a reluctance to walk one’s pet dog (5) and interventions addressing fear of falling or building confidence in mobility could benefit both dog owner and dog.

There are some limitations of this study which should be noted. Data is cross-sectional and therefore it is not possible to establish the direction of the association we identify. Longitudinal data assessing the link between dog walking and falls/mobility is not yet available. Although data are available on the number of days participants walked their dog each week, we did not have information on how much time was spent walking or the distance covered. We also did not have information on the relationship between the participant and their dog, the health status, breed, or behavior of the dog, which could all impact on levels of dog walking. The control group for the main analyses also includes participants who had a dog but did not walk with them regularly. This group was examined separately in a post hoc analysis. Variables such as falls are collected by self-report and could therefore be subject to recall bias. The proportion of participants with unexplained falls in both groups is relatively small, however, the study period was only 24 months and unexplained falls represent an important adverse outcome, with a high prevalence of injury, so even small changes in prevalence are important. Strengths include the large, well-described population-representative cohort of community-dwelling older people, and objective measures used to assess mobility.

Importantly, although we adjust for a broad range of covariates, the complex relationship between dog walking and falls/mobility (with bidirectional components) means that there may be factors other than those included in this study that impacts an older person’s ability to walk a dog regularly, and therefore the possibility of some residual confounding cannot be excluded. Further, potential pathways underpinning the relationship between dog walking and better mobility and reduced falls are unclear. Future dedicated studies are required to tease out this relationship further and adjust for additional important covariates such as the time spent dog walking.

In conclusion, this study suggests a lower prevalence of mobility impairment, falls, and fear of falling among community-dwelling older people who regularly walk their dog. Although longitudinal data confirming the direction of this association are required, as well as dedicated studies into the benefits of dog walking, older people should be encouraged to continue regularly walking their dog where feasible as it may have a role in maintaining mobility and reducing fall burden in later life.
